# Three-Dimensional-Planned Patient-Specific Guides for Scaphoid Reconstruction: A Comparative Study of Primary and Revision Nonunion Cases

**DOI:** 10.3390/jcm14062082

**Published:** 2025-03-19

**Authors:** Michael A. Wirth, Mauro Maniglio, Benedikt C. Jochum, Sylvano Mania, Ladislav Nagy, Andreas Schweizer, Lisa Reissner

**Affiliations:** 1 Department of Hand Surgery, Balgrist University Hospital, University of Zurich, 8008 Zurich, Switzerlandladislav.nagy@balgrist.ch (L.N.); andreas.schweizer@balgrist.ch (A.S.); lisa.reissner@balgrist.ch (L.R.); 2 Department of Hand Surgery, Kantonsspital St. Gallen (KSSG), Cantonal Hospital St. Gallen, 9007 St. Gallen, Switzerland; 3 Department of Hand Surgery, Centre Hospitalier Universitaire Vaudois (CHUV), University Hospital, 1005 Lausanne, Switzerland

**Keywords:** surgical planning patient-specific guides, computer-assisted 3D planning, scaphoid bone, fracture nonunion, wrist, osteotomy

## Abstract

**Background:** Scaphoid reconstruction after an established non- or malunion is challenging and recent developments have shown the feasibility to reconstruct it with 3D-planned and -printed patient-specific instrumentation. **Methods:** Our study compared the clinical outcome of computer assisted 3D-reconstructions of the scaphoid using patient-specific guides for primary and revision reconstructions of scaphoid nonunion regarding clinical outcome. Therefore, 39 patients with primary scaphoid nonunion or malunion and 15 patients with nonunion or malunion after a previous operative treatment were treated with patient-specific guides and followed up for a mean of 10.5 months. The consolidation was assessed with a CT-scan, and the time to consolidation was recorded. Pain level, satisfaction, wrist range of motion, and grip strength were measured and compared. **Results:** The wrist range of motion and grip strength of the two groups were similar, except for the wrist extension, which was significantly reduced in the revision group. Consolidation was observed in 36/39 patients (92%) in the primary group and in 13/15 patients (87%) in the revision group. Our results showed similar clinical results postoperatively between primary reconstructions and revision surgery. **Conclusions:** The use of 3D-planned and -printed patient-specific instrumentation proves to be similarly effective in revision surgeries for the reconstruction of the scaphoid as it is in primary surgeries.

## 1. Introduction

Scaphoid nonunion occurs in 5–15% of all fractures [1]. Treatment of nonunions is complicated due to a complicated three-dimensional (3D) anatomy of the scaphoid shape only visualized partially with surgical exposure, tenuous vascularity sometimes leading to avascular necrosis (1% in waist fractures and up to 40% in proximal pole fractures) [2], and fragment displacement with the typical humpback deformity [3]. Exact reconstruction of the anatomic shape of the scaphoid is the main treatment goal in addition to achieving osseous consolidation [4,5]. This is especially important because of the crucial role of the scaphoid in functionally connecting the proximal and distal carpal rows.

The surgical reconstruction of scaphoid nonunions results in relatively low revision rates of 5–6% when using non-vascularized or vascularized bone grafts [6].

Patient-specific planning and guides have been successfully used for complex corrective osteotomies in hand surgery [7,8,9,10] and more specifically in scaphoid reconstructions as well. Three-dimensional surface models of the healthy, opposite bone reconstructed from computed tomography (CT) investigations are mirrored and compared to the pathologic bone [9,11,12].

The use of patient-specific guides on the scaphoid has been shown to be significantly more precise compared to free-hand correction [3]. It offers the further advantage of preoperative planning of the screw trajectory in a way that the screw does not interfere with the canal of the screw used in the previous operation or areas of bad bone quality. Neither the union rate nor the clinical outcomes of such 3D-planned and -operated scaphoid reconstructions have been elucidated in a larger series and therefore, the effectiveness of this surgical technique remains uncertain. In addition, the use in secondary-revision surgeries after persistent scaphoid nonunions has never been exanimated nor compared to primary reconstruction of scaphoid nonunions.

The aim of this study was therefore to investigate the union rate and the clinical outcomes of 3D-computer-aided, primary and revision scaphoid reconstructions using patient-specific guides. In addition, we wanted to compare the results of primary nonunions and revision cases, exanimating if the effect and advantages of 3D-planning also led to good clinical outcomes in these more complex persistent scaphoid nonunions. We hypothesized that the use of patient-specific guides would enable surgeons to perform an accurate reduction of the fragments and therefore enhance union rates, leading to good clinical results also in revision cases.

## 2. Methods

From 2010 to 2019, a total of 54 patients with scaphoid malunion (N = 5) or nonunions (for a mean of 43 months) in the middle third (N = 47) or the junction between the middle and proximal third (N = 7) were operated by 2 senior hand surgeons in our institution using 3D-planned patient-specific guides.

Thirty-nine of these patients were surgically treated with primary scaphoid nonunions, including missed fractures, and conservatively or surgically treated fractures in the middle third or the junction between the middle and proximal third. The treatment of these fractures nonunion was defined as primary scaphoid reconstruction. In contrast to 15 patients with nonunion after a prior treatment for an already existing nonunion, previously treated with a screw fixation of the scaphoid or after reconstruction using autologous bone grafts or vascularized grafts, in this group, a second reconstructive surgery was performed and defined as a revision surgery.

Scaphoid fracture union was defined as trabecular bridging of 65% or more of the cross-sectional area, while nonunion was defined as the absence of such a union over at least 9 months. All scaphoid nonunions treated surgically for a reconstruction were included. SNAC-wrists up to Stage 1 are treated with a reconstruction in our institution. Fresh scaphoid fractures were excluded.

The clinical and radiologic outcomes were recorded included the consolidation rate and timepoint, ROM, and grip strength. These outcomes were then compared between the primary nonunions and the revision cases at the end of the follow-up.

Patients gave informed consent, and ethical approval was obtained from the responsible ethics committee (W859/BASEC No. 2020-02860).

### 2.1. Preoperative Planning

**Figure 1:**
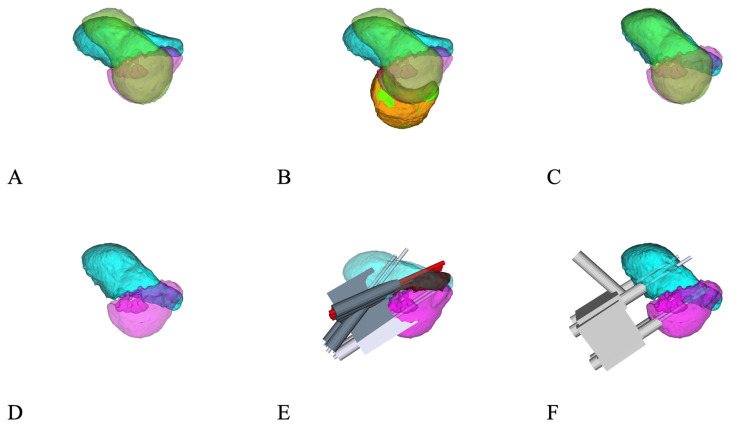
(**A**): Three-dimensional model of the scaphoid nonunion (cyan and pink) and the contralateral, healthy scaphoid (green) based on CT-scans of both wrists. (**B**): Addition of the lunate (orange) as a further reference point. (**C**): Reposition of the scaphoid nonunion to the anatomic position. (**D**): Dorsal overlap zone between the two fragments in the correct position. (**E**): Guide to place K-wires and removal of the dorsal overlap (marked in black) by drilling in the red trajectories. (**F**): Reposition guide with trunnion to fix an additional guide to aim the K-wire used to place the cannulated compression screw later.

Preoperative evaluation of the affected and the contralateral, healthy scaphoid was performed with 1 mm thick CT scan slice images (Philips Brilliance 40, Philips Healthcare, Best, The Netherlands). The raw data were segmented using Mimics software (Version 27.1; Materialise, Leuven, Belgium) to obtain 3D-surface models of both sides. These were imported into CASPA (Full version 5©, Balgrist CARD AG, Zurich, Switzerland) software. The mirrored contralateral model of the healthy side served as a template for the reconstruction of the affected scaphoid, as shown in Figure 1. This template was first aligned to the proximal pole of the non-united scaphoid via the application of the iterative closest point registration algorithm [11]. The ideal alignment was manually refined using the lunate as an additional reference point. Next, the distal pole of the scaphoid nonunion was aligned to the template. Based on the relative transformation of the distal fragment before and after alignment, a preoperative plan was created, and patient-specific guides were designed to allow for the exact execution of the plan. For this purpose, pre- and post-reduction guides were designed to fit as a palmar or dorsal scaphoid guide. Contact areas ranging from 4 × 6 mm to 6 × 8 mm were fashioned on both poles of the non-united scaphoid for the guides. Based on files provided in the stereolithography format, the guide models were produced by Medacta SA (Castel San Pietro, Switzerland) using a selective laser-sintering device. Biocompatible polyamide PA2200 was used as the raw material. All guides were verified preoperatively and tested on a true-size replica of the pathological scaphoid both pre- and post-reduction. The guides and replica were sterilized by conventional steam sterilization.

### 2.2. Surgical Technique

**Figure 2:**
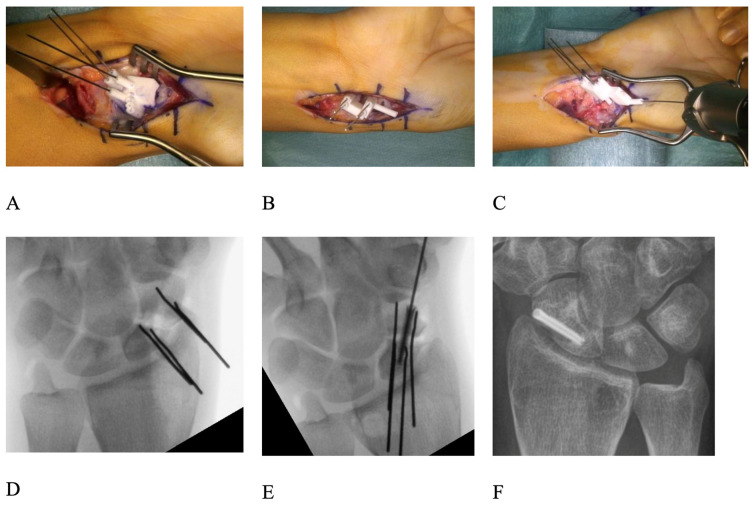
Clinical images of: (**A**): First guide to place the K-wires in the scaphoid and remove the dorsal overlap by drilling. (**B**): Reposition guide with now parallel K-wires. (**C**): Additional guide to place the guidewire for the cannulated screw. (**D**): Fluoroscopy view of the reposition. (**E**): Fluoroscopy view of the cannulated screw in situ (**F**): X-ray 6 months postsurgery with consolidation of the scaphoid.

The surgical access to the scaphoid was chosen according to the fracture location: dorsal in proximal pole fractures and palmar in waist and distal fractures. The bone surfaces of the proximal and distal pole were carefully debrided to allow for the exact positioning of the pre-reduction drilling guide. The nonunion side had to be truly debrided and cleaned to allow an exact positioning of the guide. Two parallel 0.8 mm K-wires were drilled through the planned drilling holes of the guide into both the proximal and distal fragments. The positions and directions of these four K-wires in their preoperative configurations were computed such that the proximal K-wire pair and the distal K-wire pair would later be positioned parallel to each other through the application of the post-reduction guide. Debridement of both fracture surfaces was performed until healthy, bleeding cancellous bone was encountered on both sides and, in some cases, the dorsal apposition callus was removed to ensure exact reduction [11] (Figure 1E and Figure 2B). Next, the post-reduction guide was fitted over both sets of K-wires, bringing them into parallel position and thereby reducing the scaphoid in the planned fashion, symmetric to the healthy side (Figure 1F). The parallelism of the K-wires was then double-checked by image intensifier (Figure 2D).

The trajectory of the cannulated headless compression screw (HCS) (either 2.4 mm in diameter; Synthes, Solothurn, Switzerland or 2.5 mm in diameter; Arthrex, Naples, FL, USA) used for fixation was also planned preoperatively. Using a 3D-model of the screw in the preoperative plan, care was taken so that its position would not interfere with the K-wires after reduction. In revision cases, the screw was placed in a fashion so that it would not interfere with the trajectory of the old screw from the previous operation and would be placed in an area of sufficient bone density and quality to ensure stability. Based on that model, we designed an aiming guide for the placement of the screw’s 1.1 mm guide wire which could be attached to the reduction guide (Figure 2).

A vascularized bone graft, autologous cancellous bone graft from the distal radius or iliac crest or a combination of vascularized and cancellous bone grafts were inserted between the two fragments and the fixation screw was inserted along the guidewire. The decision about the bone graft used was made by taking into account the surgical approach, the supposed vascularity of the proximal fragment, patient factors, and in the revision group, the prior surgery [13]. Whenever a bony defect was present after the nonunion debridement, a bone graft was used. If the vascularity of the proximal fragment was judged insufficient (based on radiological and intra-operative findings) a vascularized bone graft was used. A 1,2 ICSRA in case of a proximal pole fracture and a palmar carpal artery bone graft in more distal fractures were used [13]. The position of the screw and the overall reduction was then controlled with an image intensifier (Figure 2E,F. The wound was then irrigated and closed in a conventional fashion, and a forearm orthosis with inclusion of the thumb was applied for 8 weeks.

A CT-scan was performed 8 weeks postoperatively. In case of absent consolidation, immobilization with a forearm orthosis with thumb inclusion was continued, and follow-up CT-scans were scheduled every 6–8 weeks until consolidation was observed (bridging of 65% or more of the cross-sectional area). Time until consolidation was recorded. Furthermore, the range of motion (ROM) in flexion/extension and radial/ulnar deviation as well as grip strength using a Jamar Dynamometer were measured at the final follow-up and results were compared between the groups.

### 2.3. Statistical Analysis

The statistical analysis was performed using IBM SPSS, version 26 (SPSS Inc., Chicago, IL, USA).

The distribution of the data was assessed using the Shapiro–Wilk test. Homogeneity of variance was evaluated with Levene’s F-test at a significance level of 0.05. All samples showed homogeneity of the variance. To compare the clinical data from pre- to postoperation, a paired *t*-test was used. To test the differences between the postoperative results in the primary and revision group, two-tailed Student *t* tests were used for continuous data. For ordinal data, the Mann–Whitney U-test was used. The significance level was set at 0.05.

## 3. Results

In the primary group, 17 patients were treated using an autologous cancellous bone graft either from the iliac crest or the distal radius, 19 with a vascularized bone graft either dorsal (N = 7; 1,2 ICSRA/Zaidenberg graft) or palmar (N = 12; palmar carpal artery/Kuhlmann/Mathoulin graft). In three patients, no bone graft was used. For fixation, two types of headless compression screws were used. Either Synthes screws (2.4 mm in diameter; Synthes, Solothurn, Switzerland) in 24 patients or Arthrex screws (2.5 mm in diameter; Arthrex, Naples, FL, USA) in 14 others. In one patient, because of the small fragment size two K-wires (1 mm) were used for fixation.

In the revision group, 14 patients were treated using a vascularized bone graft, either dorsal (N = 1) or palmar (N = 13), and in 1 patient, no bone graft was necessary. Ten Synthes screws and five Arthrex screws were used in that group.

Mean follow-up in the primary group was 10 months (range 3–16). In the revision group, mean follow-up was 12 months (range 7–16 months). In most patients, clinical data at 12 months postoperation were analyzed, but nine patients had a shorter follow-up. Because of an excellent postoperative course with consolidation at 8 weeks and a very good clinical improvement, their cases were closed earlier. See patient demographics in Table 1.

Consolidation in the primary group was observed in 36 out of 39 (92%) patients on average after 3 months (range 2–12 months). In the revision group, in 13 out of 15 patients (87%), the average was after 5.5 months (range 2–15 months). Two patients in the primary group were followed up longer with asymptomatic nonunions until consolidation was observed after 35 and 62 months. Excluding these two outliers, the difference in time to consolidation was significant (*p* = 0.004).

The ROM of the operated wrist joint was flexion/extension 56 (SD 16)°/60 (SD 16)° radial/ulnar deviation 17 (SD 7)°/36 (SD 8)° in the primary group and flexion/extension 52 (SD 10)°/51 (SD 14)°, radial/ulnar deviation 15 (SD 6)°/35 (SD 6)° in the revision group. The wrist’s ROM of the two groups was similar for radial deviation (*p* = 0.34), ulnar deviation (*p* = 0.62), and for flexion (*p* = 0.31), except for wrist extension, which was significantly reduced (−9°) in the revision group (*p* = 0.05). There were no significant differences in grip strength with 37.1 (SD 13.4) kg (84% of the contralateral hand) in the primary group and 39.4 (SD 10.3) kg (*p* = 0.69) (85% of the contralateral hand *p* = 0.97) in the revision group. The time to consolidation and the clinical results are also summarized in Table 2.

## 4. Discussion

Early and appropriate treatment of scaphoid fractures has been shown to prevent complications, alleviate symptoms, and reduce the risk of wrist arthritis. However, these fractures are often overlooked, with up to 30% of cases going undiagnosed when evaluated using only plain X-rays [14].

The nonunion of scaphoid fractures results in various complications, such as wrist joint degeneration, which correlates with the extent of fracture displacement and the presence of carpal instability [13,15]. When nonunion persists after prior fixation attempts, it leads to a decrease in both the quantity and quality of the remaining bone, making subsequent corrective surgery more challenging [16], in addition to other obstacles like the tenuous vascularity, old screws still in place, and limited exposure [3]. Reconstruction procedures with the use of 3D-printed guides have previously been shown to result in a more accurate reduction with residual malalignment compared to the healthy side, of 6° with the use of guides and 25° with freehand corrections [3]. The importance of an anatomic reconstruction with high accuracy has been highlighted in multiple previous studies [17,18,19,20]. Even if the guides probably do not affect the bone healing directly, the improved correction affects the biomechanics of the wrist and limits the cartilage wear in the wrist, leading to a decrease in the typical SNAC-wrist changes. Still, in today’s clinical practice, plain radiographs or slices of CT scans are mostly used for planning scaphoid reconstructions [21,22,23,24], despite known limitations [25,26,27,28]. The use of guides allows for precise screw positioning in the three-dimensional plane, which is otherwise difficult, even with the use of intraoperative navigation [29].

This study reported the consolidation rates and clinical outcomes of patients treated with such 3D-planning and 3D-printed, patient-specific guides for revision reconstructions of the scaphoid, after persistent nonunion following failed reconstructions. These results were compared to a cohort of primary scaphoid reconstruction with 3D-printed and patient-specific guides. Even with these unfavorable circumstances, this reconstruction method with 3D-printed, patient-specific guides provided promising results with a good union rate of the scaphoid (13 out of 15 showed full union; 87%).

The consolidation rates in our revision group were similar to those in the primary group, despite the increased complexity of the cases. Revision surgeries for scaphoid nonunion following failed fixation remain challenging and yield highly variable outcomes, with union rates ranging from 50% up to 86%. [30,31,32,33]. Compared to the consolidation rates reported in the literature, our results lie in the upper range.

In a case series examining the use of medial femoral condyle (MFC) vascularized grafts in 16 cases of failed scaphoid fixation, an 82% union rate was achieved after a second operation [16].

Another study, which included 19 cases of failed scaphoid fixation with persistent nonunion, reported a 74% union rate after treatment with either vascularized or non-vascularized grafts [33]. A larger study involving 48 patients recorded an 82% union rate in the management of persistent scaphoid nonunion following failed fixation, using vascularized or non-vascularized grafting depending on the fracture pattern [34]. These two studies are particularly relevant for comparing with our results, as they also employed both types of grafts, suggesting that our union rate is slightly higher.

Additionally, a systematic review by Moon et al., analyzing 19 studies on revision scaphoid fixation with vascularized bone grafting in 184 cases, found an overall union rate of 86% [32].

Some authors use scaphoid plates in revision cases to account for the forementioned difficulties with high reported hardware removal rates of 65% [35], making a third surgery very likely.

In our series, good functional outcomes in ROM and grip strength were found, without a significant difference in consolidation rate, wrist flexion, or grip strength to the primary reconstructions. Only the time to consolidation was longer (5.5 vs. 3 months) and the wrist extension amplitude significantly lower (51° vs. 60°) as in primary cases. The loss of wrist extension may be a consequence of an increased amount of scar tissue formation after a second surgery. It is debatable if these 9° of extension lost are clinically relevant.

Our study limitations included the retrospective character of the study, a relatively short follow-up, the lack of control group (with conventional scaphoid reconstruction), and inhomogeneous graft choice, depending on the choice of the surgeon. On the other hand, we investigated a relatively high number of patients with scaphoid reconstructions, and all operations were performed by two senior hand surgeons with a consecutive high consistency of the quality of the operative approach.

Unfortunately, the follow-up time was too short to note possible benefits of an increased accuracy of the reconstruction, with less degenerative changes and thus better long-term pain, strength, and range of motion values. In future studies, it would therefore be interesting to schedule long-term clinical and radiological follow-up examinations of patients treated with patient-specific guides and compare them to free-hand/conventional corrections at mid- and long-term follow-up. However, the use of 3D guides has not been employed long enough to have access to long-term results yet.

## 5. Conclusions

In conclusion, the use of patient-specific, 3D-printed guides was found to be successful in the management of persistent scaphoid nonunion after a failed previous fixation. It resulted in favorable consolidation rates, comparable to primary reconstructions and in the upper range of the reported rates in the literature. In addition, the high accuracy of reconstructions led to good clinical results even in difficult revision cases. Further research comparing directly the use of 3D-printed guides and conventional reconstruction of the scaphoid is needed.

## Figures and Tables

**Table 1:** Demographic data (M = male, F = female, L = left, R = right).

Group	Age (Years)	Gender	Affected Side	Time of Nonunion (Median in Months)
Primary	29 (range 17–68, SD 12.7)	35 M, 4 F	20 R, 19 L	43 (5–406)
Revision	27 (range 18–46, SD 6.1)	15 M, 0 F	10 R, 5 L	40 (11–68)

**Table 2:** Patients’ postoperative clinical data: wrist’s ROM in degrees, grip strength measured with a Jamar Dynamometer in kilograms, time to radiologically confirmed consolidation in months.

Group	Extension	Flexion	Radial Deviation	Ulnar Deviation	Grip Strength	Grip Strength Healthy Side	Consolidation Time
Primary	60 (SD 16)	56 (SD 16)	17 (SD 7)	36 (SD 8)	37.1 (SD 13.4)	44.6 (SD 14.5)	10 (3–16) *
Revision	51 (SD 14)	52 (SD 10)	15 (SD 6)	35 (SD 6)	39.4 (SD 10.3)	46.5 (SD 9.5)	12 (7–16)

* Two outliers not included: consolidation after 35 and 62 Months.

## Data Availability

The data presented in this study are available on request from the corresponding author due to (data privacy restrictions).
